# Working memory: Differences between young adults and the aged in listening tasks

**DOI:** 10.1590/s1980-57642008dn10200006

**Published:** 2007

**Authors:** Maria Isabel d'Ávila Freitas, Ariella Fornachari Ribeiro, Márcia Radanovic, Leticia Lessa Mansur

**Affiliations:** 1 Master in Speech Pathology. Specialist in Speech Therapy - Department of Physioterapy, Speech Therapy and Occupational Therapy. Faculty of Medical Sciences of University of São Paulo.; 2 Specialist in Speech Therapy - Department of Physiotherapy, Speech Therapy and Occupational Therapy. Faculty of Medical Sciences of University of São Paulo.; 3 Researcher of Group of Cognitive and Behavioral Neurology. Division of Neurology. Hospital of Clinics - Faculty of Medical Sciences of University of São Paulo.; 4 Lecturer of Speech Therapy Course. Department of Physiotherapy, Speech Therapy and Occupational Therapy. Faculty of Medical Sciences of University of São Paulo. Researcher of Group of Cognitive and Behavioral Neurology. Division of Neurology. Hospital of Clinics - Faculty of Medical Sciences of University of São Paulo.

**Keywords:** short-term memory, language, elderly, young

## Abstract

Working memory is a system with a limited capacity which enables the temporary storage and manipulation of the information necessary for complex cognitive tasks. Numerous studies have suggested that performance in these tasks is related to age where older adults have a lesser performance than the young.

**Objective:**

To analyze the processing functions of working memory in a listening task.

**Method:**

59 educated participants aged between 19 and 76 years having no memory complaints were divided into two groups (young and aged adults). The test administered was the adapted Listening Span, in which the subject listens to a sentence, judging whether it is true or false and, concomitantly, stores the last word of each sentence for later evocation.

**Results:**

In the judgment task, performance of both groups approached to a similar average. Results of sentence recall demonstrated that with the increase in number of sentences at each level, performance of both groups declined. In the blocks of sentences 1 and 2 at level 1, all participants performed similarly. In the block of sentences 3, at level 1, there was a difference between the young and the aged. From this level onward (retention of 3 to 5 items), the aged and the young differed significantly.

**Conclusions:**

An increase in the number of sentences diminished participants’ performance of temporary storage in the recall tasks, while not interfering in the processing of sentences during judgment. The difference between the young and the aged became more accentuated as item retention demands increased.

## Abstract (pt)

A memória de trabalho é um sistema de capacidade limitada que permite o armazenamento e a manipulação temporários da informação, necessários para realização de tarefas cognitivas complexas. Vários estudos sugerem que a idade interfere no desempenho em tarefas cujo processamento solicite memória operacional e que idosos têm desempenho inferior aos jovens.

**Objetivo:**

Analisar as funções de processamento e armazenamento temporário da memória de trabalho em tarefa de escuta.

**Métodos:**

59 sujeitos escolarizados sem queixa de alteração de memória com idades entre 19 e 76 anos divididos em dois grupos (adultos jovens e adultos idosos). O teste realizado foi adaptado do Listening Span, no qual o sujeito tem que ouvir uma sentença, realizar julgamento (verdadeiro/falso) e, concomitantemente, armazenar a última palavra de cada sentença para evocá-la posteriormente.

**Resultados:**

Na tarefa de julgamento o desempenho médio dos grupos foi similar. Os resultados da evocação de sentenças demonstram que com o aumento do número de sentenças em cada nível, o desempenho dos grupos também declinou. Nas sentenças dos blocos 1 e 2 do nível 1 os participantes comportaram-se semelhantemente. Nas sentenças do bloco 3, do nível 1, existiu diferença entre jovens e idosos. A partir deste nível (retenção de 3 a 5 itens), os idosos e os jovens diferiram significantemente.

**Conclusão:**

o aumento do número de sentenças diminuiu o desempenho dos participantes no armazenamento temporário em tarefas de evocação, mas não interferiu no processamento de sentenças durante o julgamento. A diferença entre jovens e idosos se acentuou conforme o aumento na demanda de retenção.

Working memory is a system of limited capacity, able to store information temporarily for mental manipulation, and is an integral part of the human memory system.^1^

This construct^2^ underpinned the definition of the role of temporary storage of information in the performance of a wide variety of complex cognitive tasks. It is a multi-component system composed of the *central executive* which controls attentional capacity, the slave systems denominated by the *phonological loop*, responsible for acoustic and verbal information, and *visuo-spatialsketchpad* which performs a role similar to the latter, but handles visual and spatial information.^3^ Later, an episodic component (episodic buffer) was incorporated as an interface between the long and short term systems.^4^

There are various theoretical variants on working memory that differ or complement Alan Baddeley´s well-known and widely-investigated hypothesis.^5-8^ The model of working memory proposed by Baddeley is a consensus among the studies, but theoretical discussions^5-9^ over the interaction and specificity of component skills for determinate tasks, such as sequences of discourse production, is still being debated. On the relationship between working memory and language, the resource allocation model is useful to analyze the accomplishment of determinate tasks.^8^ This line considers the capacity limitations of the sub-components of working memory and is concerned with analyzing the factors associated to or determinant of this limitation, for example, age.

The preservation of working memory is crucial for the processing of language. It is essential for lexical resolution and structural ambiguity during the comprehension of sentences, reading comprehension and discourse processing.^8^

Many studies on working memory related to language have been concerned with the reading comprehension processes. An example of this type of task is one that requires subjects to read in aloud or listen to, a series of short sentences while retaining the last word of each sentence for immediate recall. In accordance with this study, the processing of information is evaluated by asking the participant simple questions about the material to be remembered (storing and processing demand), while the storing is evaluated by the accuracy of the retrieved item (storing demand only).^9^ Studies employing the approach of individual differences in working memory have correlated it to the performance on a scale of language and logic comprehension tasks, and demonstrated that the construct of working memory constitutes a useful tool for the analysis of cognitive deficits as well as for normal cognitive functioning.^10^

Among these normal processes, one focus of interest is the effect of age and ageing. There is no consensus regarding the effects of age on working memory. Researchers of working memory hold that aged adults as well as children, show shorter memory spans when compared with young adults.^11,12^ Handicaps in the aged are due to variations in the capacity to access, delete and restrict stimuli (attentional control) coupled with a decrease in information processing speed.^13^

One study has demonstrated that age differences in central executive functioning are primarily attributable to a general slowdown in the rate at which information is activated within the working memory system.^14^ However, another study revealed no such differences among younger and older subjects, in terms of the processes involved in activating operational memory.^15^

Recent studies have indicated that the predominant differences related to age are on tasks of a visuo-spatial nature^12,13^ and that isolated processing components, such as attentional operations and executive control are preserved in healthy aged.^16^

It is also known that healthy aged can minimize the overall working memory decline, although the specific process involved in this compensatory mechanism have not been entirely explained. Compensatory recruitment of attentional process was noted, especially when representational mechanisms are compromised.^16^ Modifications in attentional management (e.g. direct attention to prosodic meaning patterns) can lead to improved performance in comprehension and sentence memorization.^17^

Researchers investigating working memory skills in adults with neurologic deficits have found that these individuals present decreased working memory capacity.^18-21^ Doubts remain concerning methods of evaluating these populations, given the linguistic demands of the test and the language difficulties that overlap the working memory deficit. On the other hand, limited educational level can also interfere with the evaluation, when reading tasks are used.

The theoretical model of working memory can help us understand the breakdown in the system after a cerebral vascular accident and how this is related to language comprehension and production. Additionally, it can help in language evaluation and decisions on appropriate rehabilitation. Considering that many patients with neurological problems are elderly, it is important to verify the effect of age on working memory span.

These arguments justify the investigation of working memory listening tasks in normal elderly.

The objective of the present study was to analyze the storage functions of working memory in listening tasks.

## Methods

Fifty-nine schooled individuals (>8-year educational level), aged between 19 and 76 years and without complaints of memory, participated in this study and were divided into two groups: aged adults (AA) and young adults (YA). The YA group was composed of 38 university students from several courses while the AA group (=60 years) was composed of 21 healthy individuals, students from a Senior Citizens Education Faculty.

Those subjects agreeing to participate in the study were previously evaluated by a number of instruments (described below) in order to rule out any possibility of cognitive deficit. The exclusion criteria for the study were: hearing and/or visual loss with impact on daily functioning; below Mini-Mental State Examination normal score proposed for the Brazilian population,^22^ use of medication that could interfere with cognition; performance above the Functional Activity Questionnaire cutoff;^23^ average above the maximum of 3.41 on the Informant Questionnaire on Cognitive Decline in the Elderly -IQCODE.^24^ The cut-off criteria were defined for normality in the Brazilian population.

The test administered was an adaptation^21^ of the Listening Span,^9^ in which the subject listened to a sentence, judged whether it was true or false and, concomitantly, stored the last word of each sentence for later recall. The sentences were presented in blocks with each subsequent level having a greater number of sentences than the previous level. Each participant had to enunciate the last word of each sentence heard. The true-false component was only included to assure that the individuals processed the entire sentence and did not only concentrate on the last word.

The test was composed of 42 sentences divided into 4 levels with 3 sentence blocks each, according to difficulty (number of items to be recalled) (see appendix). The test was administered individually by the examiners (authors 1 and 2).

The performance of the participants was scored on two aspects: sentence judgement and recall of last words of these sentences. The subjects scored one point for each sentence judged correctly and one point for each word recalled correctly.

An interval of approximately 3 to 5 seconds was used between presentation of the stimulus and response of the participant.

The data were analyzed by the Mann-Whitney Test for comparisons of performance between AA and YA, according to the criteria for non-normal distribution of the data. Additionally, it sought to discriminate the predominance of influence of age or schooling factors by regression analysis, entailing application of the Poisson Log-Linear Model^25^ given it is the most adequate to analyze “count type” data.

This study was approved by the Ethics Committee of Clinicas Hospital Medical School of the University of São Paulo and all the participants signed an informed consent form.

## Results

Table 1 presents the demographic data of the participants of the study including number of participants, gender, mean age and mean schooling.

**Table 1:** Demographic data.

	Gender*		Age**	Schooling **
Group	M	F	Mean (Sd)	Mean (Sd)
Young	11	27	22.86	14.26
	(28.94%)	(71.06%)	(2.55)	(1.55)
Aged	5	16	66	11.57
	(23.08%)	(76.92%)	(4.47)	(2.85)
p	0.905		<0.0001	<0.001

* Chi-Square test;

** Mann-Whitney test.

The groups were formed with age as a reference, presenting a statistical difference between the means, as was expected (p<0.0001).

With regard to gender, there was a predominance of the female gender, both in the YA group (p=0.009), as well as the AA group (p=0.013), in equal proportion for the two groups (p=0.905).

In the judgment task, the performance of both groups approached the average score: 98.4% correct answers in AA and 99.2% in group YA. Comparison of the performance between the groups revealed no statistically significant differences in sentence judgment (Table 2).

**Table 2:** Performance according to sentence level in the recall and judgment tasks.

	Recall task				Judgment task		
Sentence level	Young Mean (Sd)	Elderly Mean (Sd)	P*		Young Mean (Sd)	Elderly Mean (Sd)	P*
Level 1 Block 1	1.97 (0.16)	1.85 (0.35)	0.091		1.94 (0.22)	2.00 (0.00)	0.739
Level 1 Block 2	1.86 (0.41)	1.66 (0.57)	0.085		2.00 (0.00)	2.00 (0.00)	1.000
Level 1 Block 3	1.97 (0.16)	1.76 (0.53)	0.030		2.00 (0.00)	1.95 (0.21)	0.763
Level 2 Block 4	2.78 (0.47)	1.90 (0.70)	<0.0001		2.97 (1.62)	3.00 (0.00)	0.868
Level 2 Block 5	2.52 (0.65)	1.81 (0.81)	0.001		3.00 (0.00)	2.95 (0.21)	0.763
Level 2 Block 6	2.89 (0.31)	2.28 (0.71)	<0.0001		2.97 (0.16)	2.85 (0.35)	0.461
Level 3 Block 7	2.55 (1.13)	1.42 (0.74)	<0.0001		4.00 (0.00)	3.90 (0.30)	0.547
Level 3 Block 8	3.15 (0.92)	2.28 (0.71)	<0.0001		3.81 (0.51)	4.00 (0.00)	0.405
Level 3 Block 9	3.28 (0.84)	2.38 (0.97)	0.001		3.93 (0.17)	3.71 (0.46)	0.071
Level 4 Block 10	3.73 (1.11)	2.23 (0.83)	<0.0001		5.00 (0.00)	5.00 (0.00)	1.000
Level 4 Block 11	3.57 (1.22)	2.33 (0.85)	<0.0001		5.00 (0.00)	5.00 (0.00)	1.000
Level 4 Block 12	3.65 (1.07)	2.0 (0.89)	<0.0001		5.00 (0.00)	5.00 (0.00)	1.000
Mean overall performance	2.83 (0.71)	1.99 (0.17)	<0.0001		3.47 (0.054)	3.44 (0.61)	0.091

The results of sentence recall demonstrate that, as number of sentences for each level increased, performance of both groups also declined. Group YA achieved an average score of 2.83 while AA obtained 1.99 (Table 2). In sentence blocks 1 and 2 of level 1 (retention of 2 items), the participants of groups AA and YA exhibited similar performance. However, in sentence block 3 of the same level, there was a difference between the young and the aged. From this level 4 (retention of 3 to 5 items), the aged and the young differed significantly.

Although the YA group had higher education than the AA group (p<0.001), the regression analysis by the log-linear model indicated that age had a greater influence in the differentiation of the groups (p<0.001) than did schooling (p=0.197) (Table 3).

**Table 3:** Regression analysis.

	Estimated	Standard	
Variable	coeffi cient	deviation	p-value *
Intercept	2.995	0.148	<0.01
Group of aged	0.309	0.061	<0.01
Schooling	0.016	0.012	0.197

* Model log-linear - Poisson.

## Discussion

The educational profile of our sample replicates that of the Brazilian Institute of Geography and Statistics (IBGE)^26^ related to schooling in different age groups. Although all the participants of the sample had been selected with 8 or more years of formal education, in the young group there was a higher concentration of high schooling. The overlapping of age and schooling effects was partially addressed in the second statistical analysis. This analysis revealed the predominance of the age effect in the sample scores and this highlighted age in the discussion of our results.

Firstly we will discuss the judgment task. This task demands the integrity of semantic memory and was introduced as a distractor of the retention task. Our results showed that the scoring of correct answers in sentence judgment had been similar in the young and aged groups.

We considered it valuable to analyze the quali-quantitative aspects of elderly behavior, due to possible contributions of semantic memory in the present study, as well as allowing observation of retention ability under isolated conditions in the short-term and without a concomitant task. This permitted the semantic-interpretive nature of the errors in the AA group to be seen. Some participants (47%) judged the sentence according to their personal experience or answered on the basis of concrete and literal meaning. Examples of this type of error follow: the participant judged the sentence *“roses have thorns”* as “false” justifying that *“some roses are cultivated not to have thorns”* or the sentence *“hens eat eggs”*, some participants stated that this was true because they said they knew that *“some hens eat the eggs”*. The sentence *“the clock tells time”*, the participant mentioned that the clock *“does not tell time, but it tells the hour”*.

For the second aspect of our discussion related to retention, young participants versus normal aged of this study presented a reduced capacity in working memory tasks. Considering that this is a listening task having a high phonological loop demand, we may state that the difference of the performance of the YA and AA groups in working memory tasks supports the notion that age restricts the processing capacity based on the phonological loop.^11^

Comparing the aged and young groups taking into account different levels of complexity of the retention task, there was no significance in the first two blocks of sentences at level 1. However, in sentence group 3 at the level 1, there was a difference between the young and the aged. Level 1 indicated retention of 2 items in the 3 blocks of sentences. Considering that the retention demand was similar for the three blocks, we assumed that the variation of behavior was due to an attentional fluctuation factor. It is likely that the aged group does not have the cognitive resources to deal with a greater demand on retention under competitive conditions. Moreover, the retention task demanded not only memorization but also active retrieval of items.

For recall, a number of strategies emerged in group AA: recalls with corrections of some semantically incorrect sentences; attempts to evoke the entire sentence, instead of the last item of the sentence as well as repetition aloud of the items to be remembered. Upon comparing correct answers in semantically correct and incorrect phrases, there was a balanced score distribution: 50.65% of errors were in true sentences and 49.35% in false sentences. These semantic strategies did not yield a greater number of correct answers in recalling the items in semantically correct phrasal contexts. Considering the demands of the task and the quali and quantitative answers, we observed that the aged displayed deficits not only in retention but also in active retrieval of items, as well as in the use of apparently preserved semantic resources, as evident in the judgment task.

Our results can be analyzed set against data in the literature data presented below. There is limited evidence that normal ageing results in notable changes in semantic memory. Generally, the few errors of our data confirm the integrity of semantic memory and retention capacity in isolated conditions. Although the total number of errors were inexpressive, almost half of participants in the AA group (47%) presented judgment errors. They used personal experience and deviated from the expected interpretation. These errors were seen in the sentences of block 9 and could explain the only statistically significant difference in this aspect of the listening judgment task. The interaction of age, working memory demand and pre-morbid verbal skill could also influence the semantic process.^27^

Judgment of sentences demands retention in non-competitive conditions. Our findings are in line with Reuter-Laurents and Jonides^16^ who observed integrity of the attentional and executive processing in tasks of retention, in isolated conditions.

The listening capacity span of our young participants was similar to that found in Daneman's^9^ pioneer study of YA university students. The span of the group varied from 2 to 4.5 (average 2.95; SD±0.72). The results of a study involving 62 participants aged between 18 and 57 years performing three tasks involving working memory, including a listening capacity task, suggested that performance of the aged is affected in verbal memory tasks, and that retrieval tasks are proportionally more challenging than recognition tasks for aged adults than for young adults.^28^ Our study is in line with the results obtained in this earlier research, whereby the aged presented poorer performance in the very recall task requiring an active information retrieval process. The possibility of central executive loss can also be considered in this task.

Studies have shown that aged adults have been less apt than the young adults to inhibit interferences made by the context of the text. Various discussions have been developed to this effect, raising the notion of participation of the central executive, whose function is to inhibit irrelevant information as well as activate and maintain the relevant information for the task.^29^ In the present study, it was not possible to broadly evaluate the role of the central executive, given the aim was restricted to the listening task. Moreover, we did not control the information relevance in the selected stimuli.

The *listening span* task requires the functioning of the phonological loop without the support of a visual-spatial sketchpad. A study on the reading comprehension task reported similar results to the listening span version,^9^ suggesting that diverse working memory tasks are inter-related and associated.^17^ By and large, differences in reading and listening are highly correlated to schooling. Schooled adults lead us to believe that these individual differences are due to language processing, and are not restricted to reading processing.

One of the verified effects in tasks needing the phonological loop is the effect of item length, which provided evidence as to the nature of the sub-vocal articulatory rehearsal process. The memory capacity for words is inversely related to the spoken duration of the words.^10^ Our test did not control the word length but rather an increasing number of items to be memorized. The participants had greater difficulty as the number of sentences increased.

It would be valuable for subsequent studies to perform reading and listening tasks that involve the visual-spatial sketchpad together with phonological tasks, in order to more broadly evaluate the multi-component system of working memory and the inter-relation of tasks. In addition increasing the sample size of schooled aged together with the inclusion of a sample of young and aged with little schooling would be useful to compare the schooling variable.

It can be concluded that increased number of sentences presented in block diminishes participants’ performance on temporary storage in recalling tasks, although does not compromise processing of sentences during judgment. Information storage in double task conditions was lower in the aged than the young adults. The difference between the young and the aged became more marked as demand for item retention increased.

## Appendix. Sample of phrases to be judged and retained.

**Table:**

**Level**	**Sample of phrases**	**Judgement**		**Recall**
Level I (3 blocks with 2 sentences each)	You sit in a chair.	T	F	chair
	Trains can fly.	T	F	fly
Level II (3 blocks with 3 sentences each)	Sugar is sweet.	T	F	sweet
	Curitiba is close to Salvador.	T	F	Salvador
	Horses run in the sky.	T	F	sky
Level III (3 blocks with 4 sentences each)	A dozen is equal to twelve.	T	F	twelve
	Bicycles are slower than cars.	T	F	cars
	A book can telephone.	T	F	telephone
	Feathers tickle.	T	F	tickle
	Babies can drive.	T	F	drive
	A clock tells time.	T	F	time
	Sky is green.	T	F	green
Level IV (3 blocks with 5 sentences each)	Carrots can dance.	T	F	dance
	Fish swim in water.	T	F	water
	We can sleep in a bed.	T	F	bed
	We lunch at night.	T	F	night
	People have eyes.	T	F	yes
	Rabbits can read.	T	F	read
	Adult birds have wings.	T	F	wings
	Chairs can eat.	T	F	eat
	Dogs have four paws.	T	F	paws
**Appendix.** Amostra das frases utilizadas para julgamento e retenção*.				
**Nível**	**Amostra de Frases**	**Julgamento**		**Evocação**
Nível I (3 blocos, cada um com 2 sentenças)	Você se senta numa cadeira.	V	F	cadeira
	Trens podem voar.	V	F	voar
Nível II (3 blocos, cada um com 3 sentenças)	O açúcar é doce.	V	F	doce
	Curitiba é perto de Salvador.	V	F	Salvador
	Cavalos correm no céu.	V	F	céu
Nível III (3 blocos, cada um com 4 sentenças)	Uma dúzia é igual a doze.	V	F	doze
	Bicicletas são mais lentas do que carros.	V	F	carros
	Um livro pode telefonar.	V	F	telefonar
	Penas fazem cócegas.	V	F	cócegas
	Os bebês podem dirigir.	V	F	dirigir
	O relógio marca o tempo.	V	F	tempo
	O céu é verde.	V	F	verde
Nível IV (3 blocos, cada um com 5 sentenças)	As cenouras podem dançar.	V	F	dançar
	Os peixes nadam na água.	V	F	água
	Podemos dormir numa cama.	V	F	cama
	Nós almoçamos à noite.	V	F	noite
	As pessoas têm olhos.	V	F	olhos
	Os coelhos podem ler.	V	F	ler
	Os pássaros adultos têm asas.	V	F	asas
	As cadeiras podem comer.	V	F	comer
	Os cachorros têm quatro patas.	V	F	patas

* The phrases in Portuguese were constructed attempting to maintain extension and lexical characteristics.
